# A 24-Year Narrative Review of an Early Intervention for Psychosis Service in Hong Kong

**DOI:** 10.1192/bjo.2025.10474

**Published:** 2025-06-20

**Authors:** Eric Chen

**Affiliations:** University of Melbourne, Melbourne, Australia

## Abstract

**Aims:** Early intervention for psychosis (EIP) programmes are specifically designed mental health initiatives aimed at the early identification and optimal management of the initial stages of psychotic disorders. The successful launch, development and consolidation of EIP programmes typically span decades and involve many factors. While quantitative meta-analytic studies have assessed the overall efficacy of EIP programmes, there remains a need for more nuanced evaluations that delve into less quantifiable processes. Such detailed accounts are crucial for facilitating the optimisation of similar programmes, especially in settings with limited resources, but they are rarely available. This study provides a comprehensive account of the development and optimisation of an early intervention programme in Hong Kong over 24 years, offering pertinent insights particularly for low- to middle-resource mental health environments.

**Methods:** A narrative account by the author, who has led the service for over 20 years. The author also has regional and international experiences in early psychosis service. Key processes involved in the service’s development, consolidation, maintenance and refinement were described. Factors that facilitate, as well as those that constrain development, were explored. The inter-relationship between these factors over time was specified.

**Results:** This long-range exploration revealed a complex network of interacting factors which both facilitated and impacted the direction and fidelity of early psychosis programmes. The network is visualised with key processes as nodes, and the mutual influence between factors as links. The evolution of this network over time is described qualitatively. Key observations involve the impact of unexpected external factors, the emergence of new data, the transition of personnel, the changing demographics, as well as relationships with other mental health programmes. The ways in which these factors interact over time are discussed.

**Conclusion:** While some programme processes can be anticipated at initial planning, many factors emerged over time and were unlikely to be addressed by an initial planning process. There are also some inherent tensions between EIP and a public mental health system. A distributed constraint satisfaction approach is proposed as a more suitable approach (than centralised pre-planning) to real-life optimisation of EIP programmes.

